# Coronary artery bypass grafting versus stents

**DOI:** 10.1186/1749-8090-8-S1-O181

**Published:** 2013-09-11

**Authors:** JCR Iglézias, A Chi, LFP Moreira, FB Jatene

**Affiliations:** 1 Cardiovascular Surgery Department, Heart Institute (InCor), Hospital das Clínicas da Faculdade de Medicina da Universidade de São Paulo, São Paulo, Brazil

## Background

Serruys P, et al [1] say that CABG is the procedure of choice for the treatment of patients with multivessel coronary artery disease resulting in lower rates of adverse clinical outcomes for cardiac and cerebrovascular diseases in the first year of follow-up. The study objectives were analyze the CABG versus stents and compare the samples studied, with respect to major cardiac outcomes.

## Methods

Study of cohort type. We analyze 202 patients undergoing CABG in the service between 17/January and 31/July/2009. The population was stratified being group G1 formed for 112 patients who received stents and group G2 formed for 90 patients undergoing to CABG. The software used was SPSS 15.0. The project was supported by FAPESP.

## Results

We observe a higher percentage of female patients in G1-49 (24%) versus 23 (11%) - P = 0.007 and found a higher percentage of diabetics in the G2-41 (20%) versus 33 (16%) - P = 0.020. There was a higher number of coronary vessels affected in G2 - 2.78 ± 1.02 versus 1.54 ± 0.74 - P = 0.001 as for the number of grafts found that was higher in the group G2- 2.71 ± 0.951 versus 1.49 ± 0.794 - P = 0.001. Of the 112 patients in G1, 72 (64.3%) received only one stent. There was a higher incidence of hospitalizing due to cardiac causes in G1 - 11 (50%) versus 3 (14%)-P = 0.006. In relation to the reappearance of angina it was higher for G1-12 (6%) versus 2 (1%)-P = 0.022. The hospital mortality was higher in G2-11 (5%) versus 5 (2%) - P = 0.064.

## Conclusion

We can state that CABG is the best procedure to treat patients with multivessel coronary disease, especially diabetics, since it allows significantly, a more complete revascularization, and decreases in the number of readmission due to cardiac causes; it reduces the recurrence of angina and improves quality of life after surgery, with similar hospital and late mortality.
